# Intramuscular Botulinum Toxin as an Adjunct to Arthrocentesis with Viscosupplementation in Temporomandibular Disorders: A Proof-of-Concept Case–Control Investigation

**DOI:** 10.3390/toxins16080364

**Published:** 2024-08-16

**Authors:** Luca Guarda Nardini, Daniele Manfredini, Anna Colonna, Edoardo Ferrari Cagidiaco, Marco Ferrari, Matteo Val

**Affiliations:** 1 Unit of Oral and Maxillofacial Surgery, Ca’Foncello Hospital, ASL 2 Marca Trevigiana, 31100 Treviso, Italy; 2 Department of Medical Biotechnologies, School of Dentistry, University of Siena, 53100 Siena, Italy

**Keywords:** temporomandibular joint, osteoarthritis, temporomandibular disorders, orofacial pain, botulinum toxin, arthrocentesis, hyaluronic acid

## Abstract

Background: The reduction in joint load is a potential beneficial factor in managing osteoarthritis of the temporomandibular joint (TMJ). This paper aims to compare the effectiveness of the intramuscular injection of botulinum toxin (BTX-A) as an adjunct to TMJ arthrocentesis plus viscosupplementation with arthrocentesis plus viscosupplementation alone in the management of TMJ osteoarthritis. Methods: A pilot clinical retrospective study examined TMJ osteoarthritis treatments. Patients were divided into two groups: Group A received BTX-A injections and arthrocentesis with viscosupplementation, while Group B received only arthrocentesis with viscosupplementation. The study assessed outcomes based on mouth opening (MO), pain at rest (PR), pain at mastication (PF), and masticatory efficiency (ME) at various time points (baseline (T0), 1 week (T1), 2 weeks (T2), 3 weeks (T3), and 4 weeks (T4)) up to 2 months after treatment. Results: The study included two groups, each with five patients. Group A received five weekly sessions of arthrocentesis plus viscosupplementation and a single BTX-A injection during the first arthrocentesis appointment. Group B underwent the five-session protocol of arthrocentesis plus viscosupplementation alone. MO, PF, PR, and ME improved quickly in T2 in both groups, but the improvement was of greater importance over the following weeks and lasted longer in Group A. Conclusions: Arthrocentesis with viscosupplementation associated with BTX-A was found to be more effective than arthrocentesis alone in improving clinical outcomes. This suggests that patients with TMJ osteoarthritis and myofascial pain may benefit from reduced muscle tone and joint load.

## 1. Introduction

The overall prevalence of temporomandibular disorders (TMDs) is approximately 31% for adults/the elderly and 11% for children/adolescents [1]. The prevalence of TMDs has increased in the last 3 years following the COVID-19 pandemic [2], as reported by Colonna et al., who showed that in a group of 506 subjects, there was a worsening of psychological status during the COVID-19 pandemic emergency, and about 50% of the subjects reported an increase in bruxism [3,4]. Specifically, 36% and 32.2% of participants reported increased pain in the temporomandibular joint (TMJ) and facial muscles, respectively, and almost 50% of the subjects also reported more frequent migraines and/or headaches [3].

TMDs are often treated stepwise. Generally, treatment starts with a combination of conservative and reversible techniques, such as anti-inflammatory drugs, muscle relaxants biopsychosocial behavioral treatments, Ref. [5], and physiotherapy and oral appliances [6]. Based on the response and the specificity of the diagnosis, escalation to more invasive interventions can be considered. In the case of temporomandibular joint degenerative joint disease (TMJ DJD), this results in alterations of the articular surfaces of the condyle and the mandibular fossa. The development of TMJ DJD is attributed to various mechanical and biological factors [7,8,9]. Patients afflicted with TMJ DJD may present with complaints of joint pain at rest and during jaw movement, restricted mouth movements, and audible joint noises such as crepitus sounds [10,11]. A conservative approach in these cases would not solve the complaints of the patient, so a second-level step may involve performing TMJ arthrocentesis with or without the positioning [12] of a steroid or hyaluronic acid (HA) into the joint [13,14,15]. The rationale for this technique is based on the fact that the concentration of HA in the synovial fluid decreases due to dilution, fragmentation, and the presence of acid molecules with lower weight than normal [16]. This compromises the conditions necessary for intra-articular homeostasis. With the growing understanding of how joint lubrication impairment can contribute to TMJ internal derangements, viscosupplementation with sodium hyaluronate, also known as HA, has become an option for managing symptoms in clinical settings [17]. The application of arthrocentesis in conjunction with HA injections has led to a progressive broadening of potential clinical indications, particularly in the context of joints afflicted by inflammatory–degenerative disorders [18,19].

As a second line of treatment for myofascial pain, one option to consider is the use of botulinum toxin injections (BTX) in the masticatory muscles [20,21,22]. BTX blocks the fusion of acetylcholine-containing vesicles to the cell membrane at the synapse, preventing the release of acetylcholine into the synaptic cleft at the neuromuscular junction. Reducing acetylcholine in the synapse leads to decreased muscle contraction after nerve signal transmission [23]. This effect is temporary, lasting about 3 months [24,25]. In addition to its well-known action on cholinergic nerve endings, such as those at the neuromuscular junction and in salivary and sweat glands, BTX-A has also been found to act on other nerve endings. It can reduce pain through both peripheral and central actions [26,27].

Major surgical treatment may be indicated in very few selected cases. Among the best-known surgical techniques, there are arthroplasty and meniscectomy in the case of advanced osteoarthritis and disc degeneration [28,29], but relapses of degenerative joint disease or ankylosis of the joint may occur with high percentages, and it may be necessary to reoperate several times [29]. Thus, even in the case of TMJ osteoarthritis, surgical techniques alone do not warrant a long-lasting resolution of the broad spectrum of musculoskeletal pain, especially considering that the articular signs and symptoms of TMJ degeneration can be triggered and/or perpetuated by prolonged muscle overload [30,31].

Within these premises, the aim of this paper is to evaluate the additional effects of muscle relaxation achieved with botulinum toxin infiltration with respect to arthrocentesis plus HA alone in the management of TMJ osteoarthritis.

## 2. Results

Ten patients were included in the study, of which three were males. The average age of Group A was 58.2 ± 13.4 years, while in Group B, it was 50.8 ± 20.9 years (Table 1).

### 2.1. Pain at Rest

**Figure 1:**
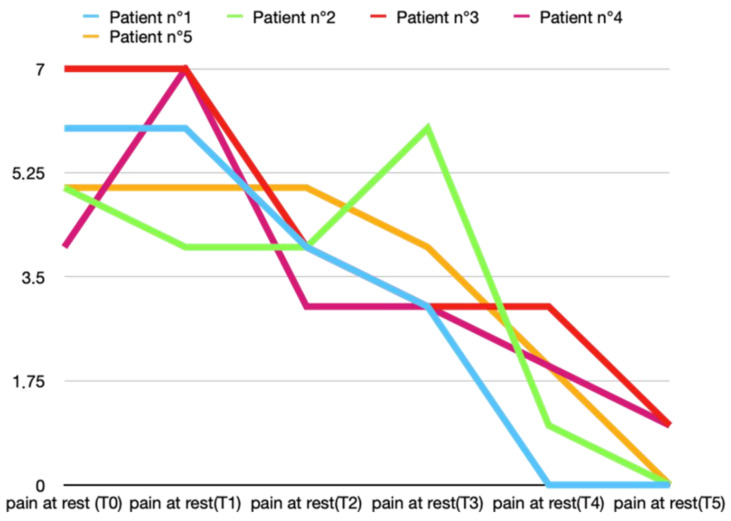
Variations in pain at rest (score according to VAS scale) in T0 (before treatment), T1, T2, T3, T4, and T5 (2 months after last arthrocentesis in T4) in Group A.

**Figure 2:**
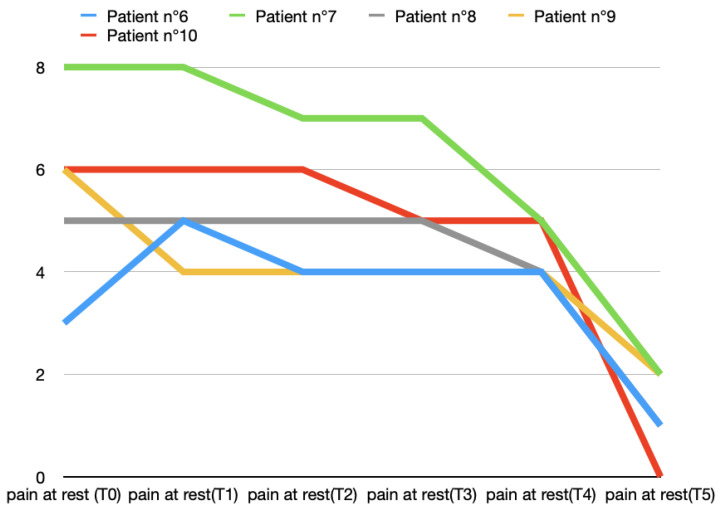
Variations in pain at rest (score according to VAS scale) in T0 (before treatment), T1, T2, T3, T4, and T5 (2 months after last arthrocentesis in T4) in Group B.

The pretreatment average pain levels at rest in Group A were 5.4 ± 1.1, and in Group B were 5.6 ± 1.8. A reduction in the mean pain at rest emerged at T2 (second week of treatment) in both groups, but in patients treated with BTX, the reduction was higher (4 ± 0.7) than in Group B (5.2 ± 1.3). The reduction in pain at rest was faster and more effective in Group A. In fact, at T5 (2 months after the end of the cycle of five arthrocentesis sessions), the mean values of pain at rest were 0.4 ± 0.5 and 1.2 ± 0.8, respectively, in Groups A and B (Figure 1 and Figure 2).

### 2.2. Pain at Chewing

**Figure 3:**
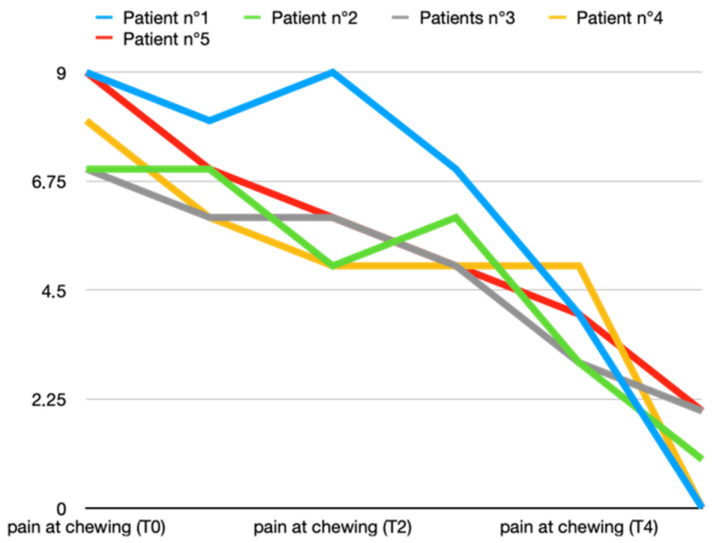
Variations in pain during chewing (score according to VAS scale) in T0 (before treatment), T1, T2, T3, T4, and T5 (2 months after last arthrocentesis in T4) in Group A.

**Figure 4:**
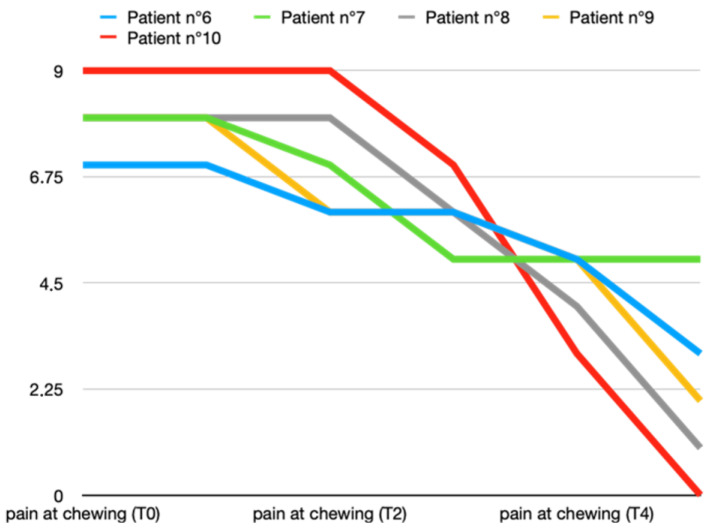
Variations in pain during chewing (score according to VAS scale) in T0 (before treatment), T1, T2, T3, T4, and T5 (2 months after last arthrocentesis in T4) in Group B.

The pretreatment average pain levels during chewing in Group A were 8 ± 1 and in Group B, 8 ± 0.7. In both groups, a reduction in average pain during chewing was obtained, but in the group that also received BTX at the first appointment, there was already a marked reduction after 7 days (T1). In both groups, the maximum reduction in pain during chewing was obtained at 2 months (T5), but in Group A, it was marked by about one point more on the VAS scale (Group A vs. Group B: 1 ± 1 vs. 2.2 ± 1.9). Figure 3 and Figure 4 show all data from both groups.

### 2.3. Masticatory Efficacy

**Figure 5:**
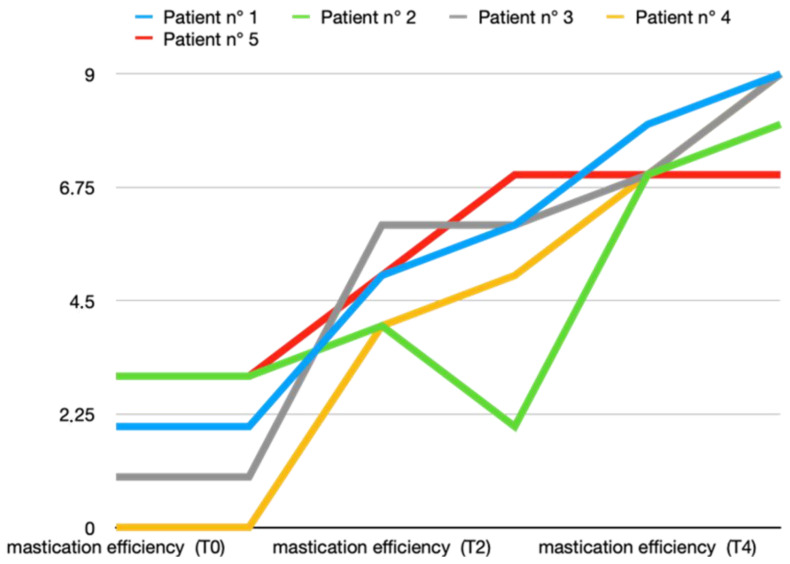
Mastication efficiency variations (score according to VAS scale) in T0 (before treatment), T1, T2, T3, T4, and T5 (2 months after last arthrocentesis in T4) in Group A.

**Figure 6:**
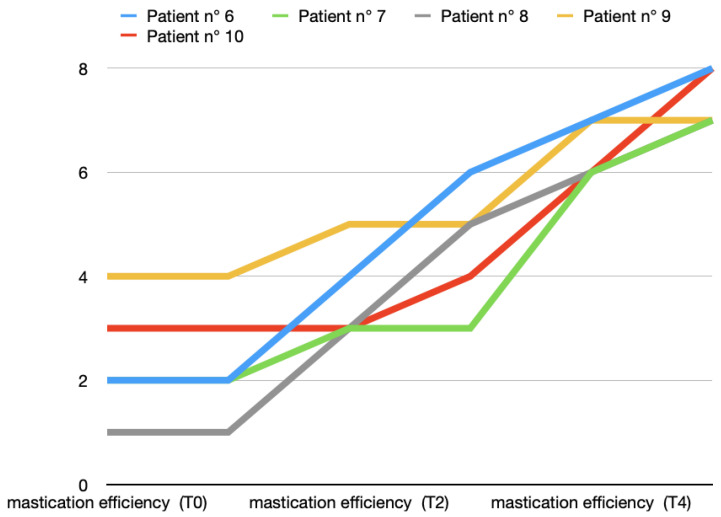
Mastication efficiency variations (score according to VAS scale) in T0 (before treatment), T1, T2, T3, T4, and T5 (2 months after last arthrocentesis in T4) in Group B.

In both groups, two months after the end of the arthrocentesis cycle, a good recovery in masticatory function was evident, with about one point in the VAS scale that differentiated between the two groups (Group A: 8.4 ± 0.9 and Group B: 7.4 ± 0.5). Figure 5 and Figure 6 show all data from both groups.

### 2.4. Functional Limitation

**Figure 7:**
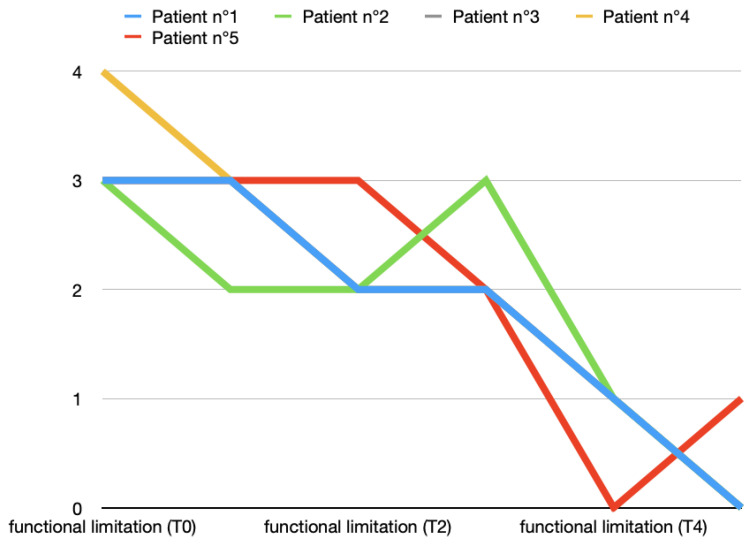
Functional limitation variations (0, absent; 1, slight; 2, moderate; 3, intense; 4, severe) in T0 (before treatment), T1, T2, T3, T4, and T5 (2 months after last arthrocentesis in T4) in Group A.

**Figure 8:**
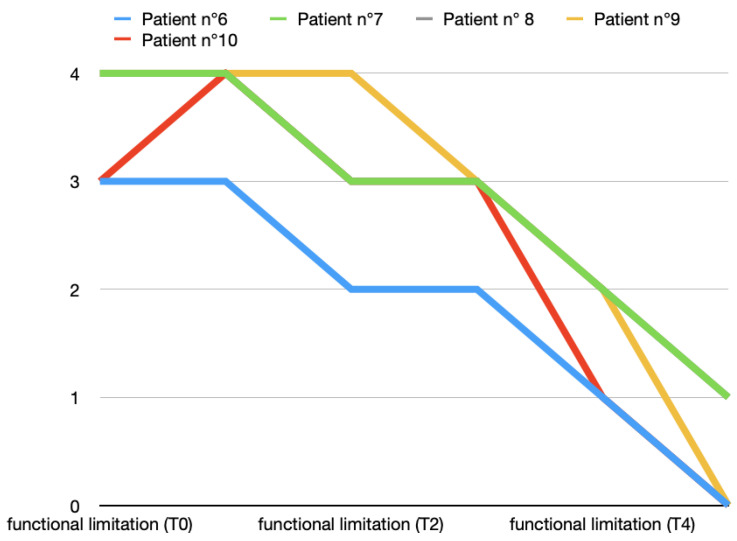
Functional limitation variations (0, absent; 1, slight; 2, moderate; 3, intense; 4, severe) in T0 (before treatment), T1, T2, T3, T4, and T5 (2 months after last arthrocentesis in T4) in Group B.

An intense functional limitation (value: 3) was highlighted in six out of the ten patients at T0, while the rest of the patients showed severe functional limitation. The functional limitation scores of the two groups were comparable at T0. This improvement was slightly faster and more constant in Group A than in Group B. Two months after the end of the arthrocentesis cycle, the functional limitation was practically superimposable between the two groups. Figure 7 and Figure 8 show all data from both groups and Table 2 highlights the average and standard deviation data.

Variations in the average and standard deviation of the functional limitation scores in T1, T2, T3, T4, and T5 are reported at the end of the table.

### 2.5. Subjective Efficacy

The subjective efficacy of the treatment at T5 was considered almost excellent (grade 4) in Group A with an average value of 3.8 ± 0.4, while in Group B, it was almost good (grade 3) with an average value of 2.8 ± 0.8.

### 2.6. Maximum Non-Assisted and Assisted Mouth Opening

Table 3 shows all the variations in the spontaneous and forced interincisal distances at T0 and T5 achieved with mouth opening. The averages and standard deviations of the MOs are also highlighted at the bottom of Table 3, which in both groups appear to be improved at T5.

### 2.7. Side Effects

One patient from Group A complained of mild transitory swallowing difficulties, which occurred 7 days post-injections but recovered within 10 days. There was no long-lasting disability. All side effects (pain in the site of injection, hematomas) in both groups resolved within 2 weeks, and there were no cases of wound infection or postoperative bleeding.

## 3. Materials and Methods

### 3.1. Ethics

The study follows the Helsinki Declaration, and the study was approved by the local ethics committee with the number “581/CE Marca”. Written informed consent was obtained from the participants.

### 3.2. Study Design/Sample

The study population was selected retrospectively within patients treated at the Unit of Oral and Maxillofacial Surgery of of Ca’Foncello Hospital (Treviso, Italy) in September 2021.

**Figure 9:**
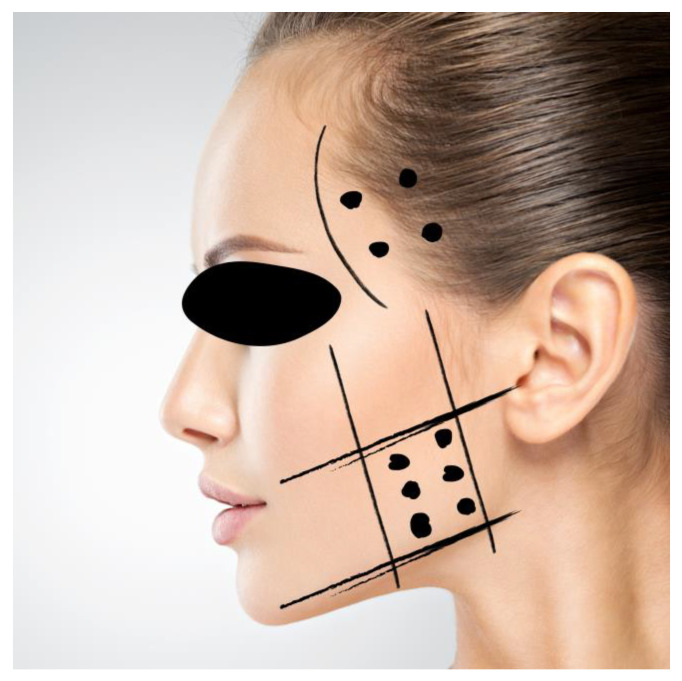
Site of injection of BTX in masseter and temporalis muscle. The dots indicate the injection sites for BTX, while the lines outline the recommended areas for injecting the masseter muscle inferiorly and the anterior portion of the temporalis muscle superiorly.

Two groups were created, comprising patients with severe joint pain due to TMJ osteoarthritis and concurrent pain upon palpation of the masseter and temporalis muscles. In both groups, a cycle of five arthrocenteses with injections plus 1 mL of hyaluronic acid (HA) 16 mg/2 mL (Synovial, IBSA FARMACEUTICI ITALIA Srl, Lodi, Italy) was provided at weekly intervals. The interventions were performed by one of two trained investigators (L.G.N.; M.V.) according to the protocol described by Guarda-Nardini et al. [32]. In Group A, botulinum toxin type A (Botox, Allergan, Inc., Irvine, CA, USA) injections were also performed during the same appointment of the first arthrocentesis. A BTX-A vial was diluted with 2 mL of 0.9% normal saline. The masseter and temporal muscles were palpated, and tender points were marked. Based on the review by Rauso et al. [33], intramuscular injections for each side (30 U) were performed using a six-point technique (5 U for each point) within the masseter muscles, and four injections (20 U) within the anterior temporalis muscles were performed bilaterally, for a treatment total of 100 U. The site of injection of BTX is shown in Figure 9.

### 3.3. Participants

Criteria for inclusion in the study were the presence of mainly arthrogenous TMD pain, with a combined diagnosis of degenerative joint disease with arthralgia lasting for more than 6 months according to the Diagnostic Criteria for Temporomandibular Disorders (DC/TMD) [34] with concurrent mild myofascial pain. Magnetic resonance imaging was used to confirm the presence of TMJ disorders in all patients. Patients were excluded if they had a prior history of TMJ treatment (e.g., conservative therapy or surgery) or BTX treatment, pregnancy, myasthenia gravis, fibromyalgia, peripheral neuropathy, or any other disorder that may interfere with neuromuscular function. Patients under the age of 18 years old were not included.

### 3.4. Variables

The following clinical parameters, based on Rosati et al. [15], were assessed at baseline and at five follow-up appointments at 1 week (T1), 2 weeks (T2), 3 weeks (T3), 4 weeks (T4), and 2 months (T5), respectively:

Pain at rest (PR) and at chewing (PC) were assessed by means of a Visual Analog Scale (VAS) from 0 to 10, with the extremes being no pain and pain as bad as the patient ever experienced;

Masticatory efficiency (ME) was assessed using a VAS from 0 to 10, the extremes of which were eating only semiliquid and eating solid hard food;

Maximum non-assisted and assisted mouth opening (MO) (in mm);

Functional limitations during usual jaw movements were subjectively evaluated by the patients, even if they had a mouth opening smaller or greater than the definition of trismus (40 mm of interincisal distance) [34]. The patients were asked to describe their sensation of mouth limitation during movements using a scale of 0, absent; 1, slight; 2, moderate; 3, intense; and 4, severe;

Subjective efficacy of the treatment (ME) (0, poor; 1, slight; 2, moderate; 3, good; and 4, excellent);

Patients were informed of the possible side effects of botulinum toxin injections (tenderness after the injection and fatigue when chewing), and each patient gave informed consent.

## 4. Discussion

In patients with degenerative TMJ disorders, arthrocentesis has been shown to be effective in managing symptoms (pain and dysfunction). Numerous techniques have been proposed to reduce the invasiveness of the treatment and improve efficacy [29,32,35,36,37]. HA infiltration after joint lavage with saline increases the potential benefit of this treatment by restoring mandibular function [38,39,40]. On the other hand, clinically, the arthrosic component is often associated with a strong muscular component, which generates joint overload and may increase pain and dysfunction. Furthermore, prolonged muscle contraction has been known to cause inflammation and localized muscular hypoxia, leading to chronic myofascial pain [41,42]. For this reason, in patients unresponsive to cognitive–behavioral therapy to reduce joint overload, it may be reasonable to associate TMJ arthrocentesis with the infiltration of BTX into the masticatory muscles [23,24,43,44].

BTX lowers TMJ load due to the reduction in muscle contractile forces. This property is obtained by the inhibition of the release of acetylcholine into the synaptic cleft at the neuromuscular junction [45]. Then, BTX acts as a modulator of central and peripheral pain transmission thanks to different neuropeptides, Refs. [46,47], which is an action that is likely to be of greater interest for the management of temporomandibular disorders.

A recent review of the literature by Delcanho et al. [23] highlighted that several randomized clinical trials show the efficacy of BTX in the modulation of pain, and in particular of myofascial syndromes, even if there are still no standardized protocols for its administration. Another systematic review and meta-analysis [48] suggested that a bilateral dose of 60–100 U could be an optimal choice for treating muscular TMD pain. Nixdorf et al. [49] highlighted a statistical significance in the maximum opening without pain (*p* = 0.02) and with pain (*p* = 0.005), with the BTX group having a relatively decreased opening. In patients treated with BTX due to myofascial pain, Guarda et al. [50] and Kutuk et al. [51] showed statistically significant improvements in lateral and protrusive jaw movements.

Due to the small number of patients included in this study, which was performed as a proof-of-concept, it was not possible to carry out a statistical evaluation, so only descriptive and comparative analyses were performed. The patients of the two groups presented overlapping PR, PC, ME, and functional limitations before treatment. Both exclusive arthrocentesis and BTX infiltration associated with arthrocentesis resulted in an improvement in all parameters. In Group A, a more rapid reduction in PR and PC and a greater improvement in ME could be seen already in the first 2 weeks (T2) compared to the treatment of arthrocentesis alone.

## 5. Conclusions

The combined use of botulinum toxin (BTX) with arthrocentesis and viscosupplementation resulted in a more rapid response compared to treatment with arthrocentesis and viscosupplementation alone. Particularly in the initial two weeks, it elicited a more substantial reduction in both pain at rest and pain at chewing. Notably, patients receiving BTX reported a quicker subjective improvement in their ability to chew. These effects are likely attributed to the alleviation of muscle overload resulting from the partial muscle paralysis induced by BTX, as well as the modulation of peripheral and central pain mediated by BTX, thereby facilitating functional relief in the TMJ and favoring the therapeutic efficacy of hyaluronic acid.

The present findings suggest that the employment of BTX and arthrocentesis with HA viscosupplementation was effective in achieving a fast and long-lasting pain reduction in patients affected by TMJ degenerative disorders. BTX infiltrations have a very fast learning method and very limited side effects and are effective in the complementary management of TMJ degenerative disorders. Further studies are needed to evaluate its routine use.

## 6. Future Perspective

The authors propose implementing a patient selection process involving targeted questionnaires (Bruxscreen [52]) to determine the suitability of candidates for BTX treatment and arthrocentesis with viscosupplementation. The objective is to enhance the effectiveness of the treatment. A future plan involves expanding the sample size through the establishment of a double-blind, randomized controlled trial to minimize potential treatment-related biases. Selected patients will be assigned to two groups: one will receive botulinum toxin infiltration and arthrocentesis with viscosupplementation, while the other will undergo infiltrations with a placebo (saline solution) and arthrocentesis with viscosupplementation. The final analysis of outcomes will incorporate data obtained from the bruxism evaluation questionnaires.

## Figures and Tables

**Table 1:** General characteristics of the two groups of patients. Legend: 0 and 1 in the TMJ column refer to the unilateral or bilateral treatment of arthrocentesis, respectively.

Groups	Patient n°	Sex	Age	TMJ
Group A	1	F	67	0
Group A	2	M	71	0
Group A	3	M	54	1
Group A	4	F	62	0
Group A	5	F	37	1
Group B	6	F	78	1
Group B	7	F	67	1
Group B	8	M	28	1
Group B	9	F	44	0
Group B	10	F	69	0

**Table 2:** Functional limitation variations (0, absent; 1, slight; 2, moderate; 3, intense; 4, severe) in T0 (before treatment), T1, T2, T3, T4, and T5 (2 months after last arthrocentesis in T4).

Groups	Patient n°	Functional Limitation (T0)	Functional Limitation (T1)	Functional Limitation (T2)	Functional Limitation (T3)	Functional Limitation (T4)	Functional Limitation (T5)
GROUP A	Average	3.2	2.8	2.2	2.2	0.8	0.2
	Standard Deviation	0.45	0.45	0.45	0.45	0.45	0.45
GROUP B	Average	3.6	3.8	3	2.8	1.6	0.4
	Standard Deviation	0.55	0.45	0.71	0.45	0.55	0.55

**Table 3:** The table shows the values of the non-assisted and assisted interincisal distance at T0 (start of treatment) and T5 (2 months after the end of treatment). At the end of the table, the variations in the average and standard deviation of the non-assisted and assisted interincisal distance are displayed at T0 (start of treatment) and T5 (2 months after the end of treatment) for Groups A and B.

Groups	Patient n°	Maximum Non-Assisted Mouth Opening (T0)	Maximum Non-Assisted Mouth Opening (T5)	Maximum Assisted Mouth Opening (T0)	Maximum Assisted Mouth Opening (T5)
Group A	1	26	43	28	46
Group A	2	34	45	38	49
Group A	3	24	38	24	41
Group A	4	18	42	25	47
Group A	5	38	45	41	46
Group B	6	21	27	23	32
Group B	7	41	44	42	47
Group B	8	20	36	27	38
Group B	9	31	34	36	38
Group B	10	15	30	20	34
GROUP A	Average	28	42.6	31.2	45.8
	Standard Deviation	8	2.88	7.79	2.95
GROUP B	Average	25.6	34.2	29.6	37.8
	Standard Deviation	10.38	6.5	9.18	5.76

## Data Availability

The original contributions presented in this study are included in the article. Further inquiries can be directed to the corresponding author.
